# Developmental Dysplasia of the Hip, Age, BMI, Place of Residence and Tobacco Abuse Increase the Odds of Aseptic Loosening in Chinese Patients

**DOI:** 10.1371/journal.pone.0085562

**Published:** 2014-01-15

**Authors:** Chuanlong Wu, Xinhua Qu, Yuanqing Mao, Huiwu Li, Fengxiang Liu, Zhenan Zhu

**Affiliations:** Department of Orthopaedics, Shanghai Ninth People's Hospital, Shanghai Jiaotong University School of Medicine, Shanghai, People's Republic of China; University of Leicester, United Kingdom

## Abstract

**Purpose:**

The purpose of this hospital-based case-control study was to evaluate the patient-related risk factors for aseptic loosening after total hip arthroplasty (THA) and total knee arthroplasty (TKA) in Chinese patients.

**Methods:**

From January 2000 to December 2012, 67 patients undergoing THA and TKA who developed aseptic loosening were detected as case subjects and 336 patients without aseptic loosening, matched by the year of index surgery and type of surgery, were selected as controls. Conditional logistic regression was used to compute odds ratios (ORs) and 95% confidence intervals (CIs).

**Results:**

The demographic factors and comorbid conditions associated with a risk-adjusted increase in aseptic loosening (in decreasing order of significance) were a rural place of residence (OR = 2.28; 95% CI: 1.21–4.30; *p* = 0.011), body mass index (BMI) ≥28 kg/m^2^ (vs. 18.5–28 kg/m^2^) (OR = 2.29; 95% CI: 1.19–4.41; *p* = 0.013), developmental dysplasia of the hip (DDH) (OR = 2.91; 95% CI: 1.11–7.66; *p* = 0.030), tobacco abuse (OR = 2.88; 95% CI: 1.05–7.89; *p* = 0.039), and age <45 years (vs. 45–65 years) (OR = 2.63; 95% CI: 1.01–6.80; *p* = 0.047).

**Conclusions:**

Patients aged <45 years and those with a BMI of ≥28 kg/m^2^, a preoperative diagnosis of DDH, history of tobacco abuse, or living in rural areas are at increased risk for aseptic loosening after THA and TKA in Chinese population. Additional systematic large-scale studies are needed to verify these results.

## Introduction

Joint replacement surgery has been universally acknowledged as one of the greatest medical advances of the 20th century. As highly successful procedures, total hip arthroplasty (THA) and total knee arthroplasty (TKA) have become conventional methods for improving quality of life and reducing pain in patients with joint disease. However, these procedures are associated with a risk of aseptic loosening, which is one of the main reasons for prosthetic failure[1]. The risk of aseptic loosening depends on a variety of factors, including, but not limited to, the patient, the surgeon, the type of implant, and the hospital [2], [3]. Most studies focus on the implant factors and surgical technique factors as the risk of PJI, however, the patient factors are fewer investigated. Patient factors mainly include demographic factors and comorbid conditions. Among demographic factors, age [4]–[6] and sex [4]–[7] have been reported as significant risk factors for aseptic loosening following THA and TKA. Only a few previously conducted studies have focused on comorbid conditions associated with the risk for aseptic loosening [8]–[10]. Body mass index (BMI) is an important factor that has been found to be significantly associated with the risk for aseptic loosening. Moreover, many studies[8]–[10] have shown that conditions including diabetes, chronic pulmonary disease, depression, alcohol abuse, drug abuse, renal disease, rheumatologic disease, and congestive heart failure were significantly associated with the risk for revision. To the best of our knowledge, there was no available data focusing on the risk factors associated with aseptic loosening in the Asian population.

Thus, we aimed to (1) evaluate the risk factors for aseptic loosening and (2) identify comorbidities and demographic factors that have not yet been reported as being associated with a significant risk for aseptic loosening after THA and TKA in Chinese patients.

## Methods

### Ethics statement

This case-control study was conducted in the Shanghai Ninth People's Hospital in Shanghai, China. It was approved by the hospital ethics committee. Written informed consent was obtained from each participant.

### Study population

From January 2000 to December 2012,69 patients undergoing THA and TKA who developed aseptic loosening were detected for the case group; two patients were excluded because of incomplete data. Our final sample comprised 67 case patients (median age, 65 years; mean age, 66 years; range, 44–87 years) and 336 controls (median age, 62 years; mean age, 61 years; range, 24–93 years) without aseptic loosening and matched by the year of index surgery and type of surgery, but not by age, sex, and other characteristics [11]. However, we attempted to control for these characteristics in the analysis. The case and control groups did not differ significantly in terms of demographic and clinical characteristics (retrieved from patients' medical records).

### Data collection

We collected the data through clinical records, including operative notes, inpatient charts, discharge summaries. The demographic characteristics of patients included age, sex, and place of residence. To investigate the influence of age on aseptic loosening, we classified the patients' ages into 4 categories (<45 years, 45–65 years, 65–75 years, >75 years) [12]. The place of residence was categorized into “rural” and “urban”. Because BMI criteria vary by country, we used the official Chinese guidelines and divided subjects into the following 3 groups: (1) below normal (≤18.5 kg/m^2^), (2) normal and overweight (18.5–28 kg/m^2^), and (3) obese (≥28 kg/m^2^).

The preoperative diagnosis for which THA (or TKA) was performed included osteoarthritis (OA), femoral head necrosis, developmental dysplasia of the hip (DDH), fracture, and rheumatoid arthritis (RA). The comorbid conditions of patients included tobacco abuse, alcohol abuse, diabetes, use of insulin for treatment of diabetes, hypertension, cardiovascular events, chronic pulmonary disease, chronic liver disease, renal disease, preoperative anemia, prostatic disorders, substance abuse, cerebral infarction, oncologic disease, neurologic disease, history of tuberculosis, gout, and ankylosingspondylitis. These comorbid conditions were based on the specific diseases that are used to determine the composite Charlson Comorbidity Index[13] as well as other diseases that are used as comorbidity measures for administrative databases and are associated with increases in length of hospital stay, hospital expenditure, complications, and mortality [14]. In addition, preexisting diseases that have been identified in clinical studies as risk factors for aseptic loosening were also included. This information was collected from patients' medical records.

### Definition of aseptic loosening

Radiographic loosening in our study was defined as: (1) signs of subsidence of >3 mm[15], (2) continuous radiolucencies at the shaft-bone or bone-cement interface or progressive radiolucencies >2 mm in the same region [16]–[18], (3) formation of multiple small cavitations or large defects around the stem [19], and (4) possible fractures of the shaft or the cement mantle[19]. Regarding progression of radiolucencies, follow-up radiographs were compared with those taken immediately after surgery to account for primarily existing radiolucent lines due to a poor cementing technique. Controls were defined as patients with a primary implant and at least 1 valid follow-up examination without any of the above-mentioned signs of loosening.

### Statistical analysis

The distributions of the demographic characteristics and comorbid conditions of patients between the case and control groups were compared using chi-square tests. Pearson's chi-square test was used for qualitative variable analysis and Fisher's exact test was used when *n* (number of data) was <20 or when any value was <5. We systematically assessed the influence of these characteristics on the risk of aseptic loosening. All tests were 2-sided. We used conditional logistic regression to calculate the odds ratios (ORs) and 95% confidence intervals (CIs) in order to estimate the effect of these factors on the risk of aseptic loosening. All variables were regressed on aseptic loosening with adjustment for the patient characteristics. All analyses were conducted using SPSS (18.0, Chicago, Illinois, USA) with significance set at the 5% level.

## Results

In Table 1, data for 403 patients who underwent total joint arthroplasty (TJA), including 67 patients diagnosed with aseptic loosening, were examined. Among the 336 patients without aseptic loosening (control group), the proportions of the prevalent demographic factors were as follows: male patients, 42.9% (144); patient age >65 years, 45.0% (151); rural place of residence, 25.0% (84); and history of THA, 70.8% (238). Among the 67 patients diagnosed with an aseptic loosening (case group), the proportions of the same demographic factors were as follows: male patients, 43.3% (29); patient age >65 years, 41.8% (28); rural place of residence, 41.8% (28); and history of THA, 61.2% (41).In the case group, the diagnoses for which the surgery was performed included OA (14.9%), femoral head necrosis(11.9%), DDH (23.9%), fracture (37.3.4%), RA(7.5%), and other diagnoses (4.5%), while the respective percentages in the control group were 21.4%, 12.8%, 15.2%, 36.6%, 8.0%, and 6.0%.There were no significant differences in the age, sex, type, and diagnosis between the 2 groups (age, *p* = 0.072; sex, *p* = 0.949; type, *p* = 0.119; diagnosis, *p* = 0.552), while the place of residence was found to be statistically significant (*p* = 0.005).

**Table 1:** Patient demographics and other characteristics according to aseptic loosening status.

	Aseptic loosening		
	Yes	No	*P* *
**Age**			0.072
**<45**	10(14.9%)	21(6.3%)	
**45**–**65**	29(43.3%)	164(48.8%)	
**65**–**75**	20(29.9%)	92(27.4%)	
**≥75**	8(11.9%)	59(17.6)	
**Gender**			0.949
**Male**	29(43.3%)	144(42.9%)	
**Female**	38(56.7%)	192(57.1%)	
**Place of residence**			0.005
**Rural**	28(41.8%)	84(25.0%)	
**Urban**	39(58.2%)	252(75.0%)	
**Type**			0.119
**THA**	41(61.2%)	238(70.8%)	
**TKA**	26(38.8%)	98(29.2%)	
**Diagnosis**			0.552
**Osteoarthritis**	10(14.9%)	72(21.4%)	
**Femoral head necrosis**	8(11.9%)	43(12.8%)	
**Developmental dysplasia of the hip**	16(23.9%)	51(15.2%)	
**Fracture**	25(37.3%)	123(36.6%)	
**Rheumatoid arthritis**	5(7.5%)	27(8.0%)	
**Other diagnoses**	3(4.5%)	20(6.0%)	

: X^2^ test for the variables; THA: total hip arthroplasty; TKA: total knee arthroplasty.

In the case group, there was a higher proportion of patients with a BMI of≥28 kg/m^2^ (50.7% vs. 33.3%, *p* = 0.024), and a higher prevalence of tobacco abuse (14.9% vs. 6.8%, *p* = 0.028) (Table 2). Table 2 shows a comparison of other characteristics between the case and the control groups; the differences in these characteristics were not statistically significant (*p*>0.05).

**Table 2:** Patient comorbid conditions according to aseptic loosening status.

	Aseptic loosening		
	Yes	No	*P* *
**BMI**			**0.024**
**<18.5 kg/m^2^**	1(1.5%)	10(3.0%)	
**18.5**–**28 kg/m^2^**	32(47.8%)	214(63.7%)	
**≥28 kg/m^2^**	34(50.7%)	112(33.3%)	
**Tobacco abuse**	10(14.9%)	23(6.8%)	**0.028**
**Alcohol abuse**	9(13.4%)	24(7.1%)	0.086
**Renal disease**	9(13.4%)	30(8.9%)	0.255
**Anemia**	5(7.5%)	40(11.9%)	0.292
**Cerebral infarction**	5(7.5%)	18(5.4%)	0.498
**Prostatic disorders**	4(6.0%)	28(8.3%)	0.514
**Oncologic disease**	4(6.0%)	14(4.2%)	0.514
**Hypertension**	18(26.9%)	78(23.2%)	0.522
**History of tuberculosis**	3(4.5%)	10(3.0%)	0.525
**Chronic liver disease**	5(7.5%)	20(6.0%)	0.640
**Neurologic disease**	4(6.0%)	16(4.8))	0.678
**Substance abuse**	6(9.0%)	26(7.7%)	0.737
**Chronic pulmonary disease**	6(9.0%)	34(10.1))	0.771
**Treatment of diabetes(insulin)**	2(3.0%)	8(2.4%)	0.772
**Gout**	4(6.0%)	22(6.5%)	0.861
**Cardiovascular events**	8(11.3%)	38(11.9%)	0.882
**Diabetes**	6(9.0%)	32(9.5%)	0.884
**Ankylosingsporidylitis**	3(4.5%)	14(4.2%)	0.908

: X^2^ test for the variables; BMI: body mass index.

Among the demographic factors, patients aged <45 years had a 2.63 times greater risk (95% CI: 1.01–6.80; *p* = 0.047) of aseptic loosening compared to patients aged 45–65 years. The other age groups (65–75 years and >75 years) had no significant association with the risk of aseptic loosening (*p*>0.05). In addition, living in rural areas conferred a 2.28 times greater risk (95% CI: 1.21–4.30; *p* = 0.011) of aseptic loosening compared to living in urban areas.

In the comorbid conditions, BMI of >28 kg/m^2^was associated with a 2.29 times increased adjusted risk (95% CI: 1.19–4.41; *p* = 0.013) of aseptic loosening compared to a BMI of 18.5–28 kg/m^2^. However, a BMI of <18 kg/m^2^ was not a significant risk factor for aseptic loosening (*p*>0.05). Patients with a preoperational diagnosis of DDH had a 2.91 times greater risk (95% CI: 1.11–7.66; *p* = 0.030) of aseptic loosening compared to patients with OA. The other preoperational diagnoses including femoral head necrosis, DDH, fracture, RA and the others were not significant risk factors for aseptic loosening (*p*>0.05).Tobacco abuse was associated with a 2.88 times increased adjusted risk (95% CI: 1.05–7.89; *p* = 0.039) of aseptic loosening. We did not find any significant difference in other factors between the cases and the controls (*p*>0.05) (Table 3). In Table 4 and Table 5, we have summarized the data of previous studies that have examined patient-related risk factors for aseptic loosening in patients who have undergone joint arthroplasty.

**Table 3:** Independent risk factors for aseptic loosening after multivariate regression analysis*.

Patient demographics and comorbid conditions	Reference	Odds Ratio	95% CI		*p* -Value
**Place of residence (Rural)**	Urban	2.28	1.21	4.30	**0.011**
**BMI (≥28 kg/m^2^)**	BMI(18.5–28 kg/m^2^)	2.29	1.19	4.41	**0.013**
**Developmental dysplasia of the hip**	Osteoarthritis	2.91	1.11	7.66	**0.030**
**Tobacco abuse**		2.88	1.05	7.89	**0.039**
**Age (<45 years)**	Age(45–65 years)	2.63	1.01	6.80	**0.047**
**Renal disease**		1.96	0.78	4.94	0.153
**Oncologic disease**		2.30	0.59	9.00	0.234
**Substance abuse**		1.73	0.58	5.18	0.328
**Ankylosingsporidylitis**		2.04	0.48	8.66	0.333
**Hypertension**		1.41	0.63	3.12	0.401
**Fracture**	Osteoarthritis	1.45	0.59	3.57	0.415
**Cerebral infarction**		1.66	0.47	5.95	0.434
**Chronic pulmonary disease**		1.49	0.53	4.14	0.449
**THA**	TKA	0.79	0.42	1.50	0.475
**History of tuberculosis**		1.59	0.35	7.27	0.550
**Anemia**		0.71	0.23	2.22	0.561
**Prostatic disorders**		0.71	0.20	2.52	0.592
**Age (65**–**75 years)**	Age(45–65 years)	1.21	0.58	2.50	0.612
**Cardiovascular events**		1.29	0.47	3.57	0.620
**Alcohol abuse**		1.28	0.49	3.41	0.626
**Treatment of diabetes (insulin)**		1.66	0.20	13.63	0.635
**Femoral head necrosis**	Osteoarthritis	1.28	0.41	3.94	0.673
**Chronic liver disease**		1.25	0.38	4.10	0.709
**BMI (<18.5 kg/m)**	BMI(18.5–28 kg/m^2^)	0.76	0.08	6.85	0.805
**Age (>75 years)**	Age(45–65 years)	1.14	0.43	3.04	0.794
**Neurologic disease**		1.18	0.30	4.57	0.816
**Rheumatoid arthritis**	Osteoarthritis	1.17	0.33	4.22	0.809
**Other diagnoses**	Osteoarthritis	1.20	0.24	6.04	0.823
**Diabetes**		1.10	0.31	3.90	0.885
**Gout**		0.93	0.27	3.30	0.913
**Male gender**	Female Gender	1.01	0.54	1.90	0.982

BMI: body mass index; THA: total hip arthroplasty; TKA: total knee arthroplasty.

Adjusted for age, sex, BMI, place of residence, diagnosis, and all comorbid conditions.

**Table 4:** Data from previous studies examining patient-related risk factors for aseptic loosening in patients who have undergone hip and knee arthroplasty.

Author	Year	Country	Research Type	Total patient number	Position	Statistical method
**Streit et al**	2013	Germany	Cohort study	326	THA- uncemented tapered titanium stem	Multivariate Cox regression
**Corten et al**	2011	Canada	Randomized controlled trial	250	THA-cemented fixation	Multivariate Cox regression
**Thillemann et al**	2010	Denmark	Case-control	4,698	THA	Logistic regression
**Hailer et al**	2010	Sweden	Cohort study	170,413	THA	Multivariate Cox regression
**Pedersen et al**	2010	Denmark	Cohort study	57,575	THA	Poisson regression analyses
**Zwartele et al**	2008	The Netherlands	Cohort study	223	THA	Multivariate Cox regression
**Haverkamp et al**	2007	The Netherlands	Cohort study	411	THA-cemented fixation	Multivariate Cox regression
**Flugsrud et al**	2007	Norway	Cohort study	1,535	THA	Multivariate Cox regression
**Bordini et al**	2007	Italy	Cohort study	4,750	THA	Multivariate Cox regression
**Münger et al**	2006	Switzerland	Case-control	5,035	THA	Logistic regression
**Malik et al**	2004	UK	Cohort study	224	THA	Logistic regression

**Table 5:** Detailed data from previous studies examining patient-related risk factors for aseptic loosening in patients who have undergone hip and knee arthroplasty.

	Streit et al(2013)	Corten et al(2011)	Thillemann et al (2010)	Hailer et al (2010)	Pedersen et al(2010)	Zwartele et al (2008)	Haverkamp et al (2007)	Flugsrud et al (2007)	Bordini et al (2007)	Munger et al (2006)	Malik et al (2004)
Patient Demographics and Comorbid Conditions	HR (95%CI), *p*-value	HR (95%CI), *p*-value	HR (95%CI), *p*-value	RR(95% CI), *p*-value	RR(95% CI), *p*-value	HR (95%CI), *p*-value	HR (95%CI), *p*-value	HR (95%CI), *p*-value	HR (95%CI), *p*-value	OR (95%CI), *p*-value	OR (95%CI), *p*-value
**Age(years)**	**Per 1 year decrease**: 1.00, (0.93–1.07), 0.91	**<65: All aseptic revisions**: 3.21, p <0.001; **Aseptic acetabular cup revisions**: 9.39, p = 0.035; **Aseptic femoral stem revisions**: 2.54, p = 0.001; **Aseptic liner/head Exchanges**: NS.		**50**–**59 vs. <50**: 0.7(0.6 –0.8), <0.001; **60**–**75 vs. <50**: 0.4(0.4 –0.5), <0.001; **>75 vs. <50**: 0.2(0.2–0.2), <0.001				**Per 5 years: Men**: Acetabular component: NS; Femoral component: NS. **Women**: Acetabular component: NS; Femoral component: NS.	**Cup failure: >70 vs. 40**–**70**: 0.66(0.41–1.08), 0.098; **<40 vs. 40**–**70**: 2.02 (1.21–3.38), 0.007; **stem failure**: NS.	**60**–**70 vs. <60**: 0.83, (0.67–1.04), 0.1; **71**–**80 vs. <60**: 0.61, (0.47–0.79), <0.001; **>80 vs. <60**:0.39, (0.21–0.71), <0.01; **Risks decreased per additional year of intervention postponement**: 0.98(0.97–0.99), <0.001	
**Gender**	**Male vs. female**: 1.54 (0.54–4.42), 0.42	**Male vs. female: All aseptic revisions**: 1.83, p = 0.011; **Aseptic acetabular cup revisions**: NS; **Aseptic femoral stem revisions**: NS; **Aseptic liner/head Exchanges**: NS.		**Female vs. male**: 0.7 (0.6 –0.7), <0.001					**Cup failure**: NS**. Stem failure**: Male vs. female: 2.00 (1.28–3.12), 0.002	**Female vs. male**: 0.64(0.52–0.78), <0.001	
**Diagnosis**				**RA vs. OA**: 1.1(1.0 –1.2), 0.2; **Fracture vs. OA**: 1.2(1.1 –1.4), 0.02; **Pediatric vs. OA**: 1.5(1.3 –1.8), <0.001; **Osteonecrosis vs. OA**: 1.3(1.1 –1.5), 0.01; **Posttraumatic OA vs. OA**: 1.5(1.0 –2.2), 0.05; **Tumor vs. OA**: 1.4(0.5 –3.7), 0.5; **Other secondary arthrosis**: 0.7(0.4 –1.0), 0.03		RA vs. OA: NS.			**Cup failure: Sequelae of congenital and pediatric diseases vs. OA**: 2.32 (1.49–3.62), 0.0001; **Femoral neck fracture and sequelae vs. OA**: 1.98 (1.24–3.17), 0.004; **Others vs. OA**: NS. **Stem failure**: Femoral neck fracture and sequelae vs. OA: 1.84 (1.09–3.12), 0.02	NS.	
**BMI (kg/m^2^)**							NS.			**25–30 vs. <25**: 1.10(0.90–1.35), 0.4; **>30 vs. <25**: 1.26(0.98–1.63), 0.08; **Risks increased per additional unit of BMI**: 1.03(1.00–1.05), 0.02	
**Diabetes**					1.02 (0.74-1.39) NS.						
**Tobacco abuse**											NS.
**Substance Abuse**			**Postoperative statin usage**: 0.38 (0.28–0.53), <0.05								**Usage of NSAID**: NS.

NS: Not significant; Blank space: information not reported; THA: total hip arthroplasty; TKA: total knee arthroplasty.

THA: total hip arthroplasty; TKA: total knee arthroplasty.

## Discussion

Our study showed that age<45 years, a rural place of residence, BMI>28 kg/m^2^, a preoperational diagnosis of DDH, and tobacco abuse were independently associated with an increased risk of aseptic loosening in patients who underwent TJA. Previous studies have found that sex (male vs. female) [4]–[7], and the preoperative diagnosis (fracture vs. OA [5], osteonecrosis vs. OA [5], femoral neck fracture and sequelae vs. OA [7])are significantly associated with an increased risk of aseptic loosening (Table 5). However, we did not find these conditions to be associated with a significant risk after controlling for all clinical and demographic factors (*p*>0.05).

### Demographic factors

We found that patients aged <45 years had a 2.63 times greater risk for aseptic loosening compared to patients aged 45–65 years. Similarly, Corten et al [4] reported that patients aged >65 years have a 3.21 times greater risk of aseptic loosening in all aseptic revisions of THA. After evaluating 170,413 THA operations from the Swedish Hip Arthroplasty Register, Hailer et al [5] found that patients aged 50–59, 60–75, and >75 years had a 0.7, 0.4, and 0.2 times, respectively, lower risk of aseptic loosening compared to patients aged<50 years. In addition, Münger et al [6] also suggested that younger age was associated with an increased risk of aseptic loosening. Other studies have reported that age had no significant effect on the risk of aseptic loosening [20], [21]. In a cohort study of 1,535 individuals of THA, Bordini et al [7] found that patients aged <40 (vs. 40–70) years have a 2.02 times greater risk of aseptic loosening in revisions of cup failure; however, data from revisions of stem failure had no statisticalsignificance.

To the best of our knowledge, no studies have specifically reported place of residence to be a risk factor associated with the incidence of aseptic loosening. We found that patients living in rural areas had a 2.28 times greater risk for aseptic loosening compared to those living in urban areas. This may be due to a higher physical activity level, delayed diagnosis of the underlying disease, irregular treatment, or financial difficulties in some rural areas.

### Comorbid conditions

Our data showed that a BMI of >28 kg/m^2^ conferred a 2.29 times higher risk for aseptic loosening compared to a BMI of 18.5–28 kg/m^2^. Similar to the results of this study, Munger et al [6], after reviewing 5,035 patients who had undergone THA, found that the risk for aseptic stem loosening increased 1.03 times per additional unit of BMI. However, they did not find any statistical significance when they compared the risk in patients aged 25–30 years with those aged <25 years and in patients aged >30 years with those aged <25 years. On thecontrary, Haverkamp et al [22] indicated that there was no association between obesity and an increased risk of aseptic loosening among THA patients.

DDH is one of the main causes of THA in young adults. In the current study, patients with a preoperative diagnosis of DDH were found to have a greater risk for aseptic loosening compared to patients with a preoperative diagnosis of OA. As mentioned above, an evaluation of 170,413 THA operations conducted by Hailer et al [5] showed that the incidence of aseptic loosening was 1.5 times higher in patients with pediatric hip diseases compared to those with OA. After a multivariate survival analysis of 4750 patients who had undergone THA from 1995 to 2000, Bordini et al [7] showed that the sequelae of congenital and pediatric diseases were independent risk factors for aseptic loosening (adjusted OR = 2.32; 95% CI: 1.49–3.62).

Though various studies have evaluated the negative effects of tobacco on surgical outcomes [23], [24], and it is generally accepted that recovery time and complication rates are increased in these patients, very few studies have examined or found tobacco abuse to be associated with an increased risk of aseptic loosening in TJA patients. Kapadia et al [25] found that alcohol abuse conferred a higher risk for revision in a 4-year study of patients who had undergone TKA. We found that tobacco abuse was associated with a 2.88 times higher risk for aseptic loosening. On the contrary, Malik et al [26] did not find a significant association between tobacco abuse and an increased risk of aseptic loosening among patients who underwent THA.

### Strengths and limitations of the study

Our study has several strengths over previously conducted studies. First, to the best of our knowledge, this is the first case-control study to investigate the association of patients' factors with the risk for aseptic loosening in the Chinese population. Second, we found that patients living in rural areas had an increased risk for aseptic loosening compared to patients living in urban areas. This will help increase awareness and serve as a forewarning for rural patients and the local government regarding this issue. Third, we found that the preoperative diagnosis of DDH was an important risk factor for aseptic loosening.

Nevertheless, this study has some limitations. First, we did not differentiate the type of implant and the site of the loosening (cup or stem). This may have affected the accuracy of our results. Second, the number of patients in this study with aseptic loosening was rather small, which may reduce the generalizability of our conclusions. Finally, >90% of the patients with diabetes had the type 2 variant, which is believed to have multiple causes (including obesity). We were unable to discriminate between type 1 and type 2 diabetes in our analyses; hence, we could not analyze the association between the diabetes subtype and the risk for aseptic loosening.

### Conclusions

In conclusion, Chinese patients aged <45 years and those with a BMI of ≥28 kg/m^2^, a preoperational diagnosis of DDH, history of tobacco abuse, or living in rural areas are at an increased risk for aseptic loosening following TJA. Further systematic studies that evaluate the risk factors for aseptic loosening in the Asian population are needed to confirm the findings of the present study.
